# Emotions' Impact on Viewing Behavior under Natural Conditions

**DOI:** 10.1371/journal.pone.0052737

**Published:** 2013-01-09

**Authors:** Kai Kaspar, Teresa-Maria Hloucal, Jürgen Kriz, Sonja Canzler, Ricardo Ramos Gameiro, Vanessa Krapp, Peter König

**Affiliations:** 1 Institute of Psychology, University of Osnabrück, Osnabrück, Germany; 2 Institute of Cognitive Science, University of Osnabrück, Osnabrück, Germany; 3 Department of Neurophysiology and Pathophysiology, University Medical Center Hamburg-Eppendorf, Hamburg, Germany; National University of Singapore, Singapore

## Abstract

Human overt attention under natural conditions is guided by stimulus features as well as by higher cognitive components, such as task and emotional context. In contrast to the considerable progress regarding the former, insight into the interaction of emotions and attention is limited. Here we investigate the influence of the current emotional context on viewing behavior under natural conditions.

In two eye-tracking studies participants freely viewed complex scenes embedded in sequences of emotion-laden images. The latter primes constituted specific emotional contexts for neutral target images.

Viewing behavior toward target images embedded into sets of primes was affected by the current emotional context, revealing the intensity of the emotional context as a significant moderator. The primes themselves were not scanned in different ways when presented within a block (Study 1), but when presented individually, negative primes were more actively scanned than positive primes (Study 2). These divergent results suggest an interaction between emotional priming and further context factors. Additionally, in most cases primes were scanned more actively than target images. Interestingly, the mere presence of emotion-laden stimuli in a set of images of different categories slowed down viewing activity overall, but the known effect of image category was not affected. Finally, viewing behavior remained largely constant on single images as well as across the targets' post-prime positions (Study 2).

We conclude that the emotional context significantly influences the exploration of complex scenes and the emotional context has to be considered in predictions of eye-movement patterns.

## Introduction

In everyday life visual attention is guided by stimulus features, often labeled as the bottom-up way of attention control, as well as by individual cognitive components, such as motivation, knowledge, and emotional status that affect attention processes in a top-down manner.

The stimulus-dependent, bottom-up component of overt visual attention, the process of actively directing the gaze, has been widely studied by detecting, analyzing, and predicting the correlations between fixation behavior and image features [1], [2], [3], [4]. However, this type of analysis mostly focuses on low-level features and geometrical properties. As a result, the variance explained is limited [5], [6]. Furthermore, the causal influence of low-level features on the selection of fixated regions is at the center of a continuing controversy [5], [7], [8], [9], [10]. Furthermore, it has been demonstrated that cognitive influences can override the stimulus saliency and thereby limit its predictive capability 11,12. A hierarchical model preferring cognitive top-down influences, if present [13], or a combination of both stimulus-driven and cognitive high-level aspects [14], [15], [16], emphasizes the need to consider top-down influences on attention processes. Such research on the impact of higher cognitive functions on attention concentrates on visual search, memory, or previous knowledge [9], [15], [17], [18], [19], [20], [21], [22], [23], [24], [25]. Remarkably, investigations on the impact of the emotional state as an integral part of cognition [26] on visual perception are rare.

The International Affective Picture System (IAPS) is a set of natural visual stimuli with validated emotional valence and arousal [27], [28], [29], [30]. It is a most appropriate tool to study visual processing in the context of emotionally relevant stimuli in clinical [31], [32], [33] and non-clinical samples [34], [35]. Nummenmaa, Hyönä, and Calvo [30] found a higher probability to fixate on emotional pictures first when presented simultaneously with neutral stimuli, which is in line with the results of Calvo and Lang [28], [29]. Face-perception studies substantiate these results. Schubö, Gendolla, Meinecke, and Abele [35] demonstrated that threatening faces were detected more quickly than friendly ones, supporting the notion of a threat-detection advantage. Interestingly, the perception of emotional faces is not simply predictable on the basis of the features of the presented stimuli, but the emotional state of the individual is important as well. Depressed patients, for instance, correctly identify sad and angry faces with a lesser intensity of facial expression than do subjects without psychopathological symptoms [32]. The impact of the subjects' emotional state on attention processes was impressively shown in a study with arachnophobic patients [36]: viewing behavior was not explicitly based on stimulus saliency and general emotional relevance of the stimuli. Rather, the subjects' emotional state, in this case spider-phobia, was the crucial factor for an increased attention to suddenly appearing objects (spiders) in the scene. Similar results were obtained with snake-phobic patients [37]. Thus, available results indicate that emotionally relevant stimuli capture attention rapidly, and visual attention is guided by emotional processes even outside of the context of emotional stimuli.

These results raise the questions to what extent viewing behavior is affected by the emotional content of complex scenes, and whether the current emotional context influences human viewing behavior under natural conditions. These two aspects have been completely neglected in past eye-tracking studies on emotion and attention. Actually, the impact of emotional priming on the viewing behavior of complex, neutral scenes has not been directly addressed. Instead, some studies that did not apply eye-tracking technology offer interesting references to emotional priming: Flaisch, Stockburger, and Schupp [38] studied changes in event-related potentials (ERP) while watching emotional primes and neutral target pictures, and they found a reduced, late positive potential in both primes and targets. Hence, psycho-physiological activation due to emotional stimuli seems to influence perception of neutral stimuli as well. However, Most, Chun, Widders, and Zald [39] also found an interference effect of emotional and perception processes, supporting the notion of prolonged processes in target perception. Consequently, when the emotional state affects neutral target processing, the rate of eye movements may increase or decrease.

Here we investigate the potential impact of the emotional context on viewing behavior. For this purpose we measured eye-movement data of subjects viewing emotion-laden as well as neutral complex scenes in different free-viewing conditions. As an important cognitive modulator, we manipulated the emotional state of the participants by using the standardized pictures of the IAPS as primes [27]. We provide information about subjects' viewing behavior by analyzing the duration of fixations, saccade amplitudes, and the spread of fixation distributions by means of a progressive entropy analysis. According to the main null hypothesis, we expected the measured parameters to remain stable across the various trials. Alternatively, if the emotional context influenced free-viewing behavior in a top-down manner, the measured eye-movement parameters should be affected.

In order to scrutinize these research questions we conducted two studies: in Study 1 we investigated whether (1) negative, neutral, or positive complex scenes elicit discriminative viewing behavior, and (2) whether the kind of emotional context constituted by these scenes affects how people observe nature landscape images. In Study 2, we implemented a more sophisticated design to (1) replicate the findings of Study 1, (2) enlarge the findings on neutral target images differing in gist, and (3) enable a comparison between the data of the present study and one of our previous studies [21]. The latter study serves as the baseline condition in which subjects observed images under the same natural conditions, but without an emotional context

## Study 1

### Methods

#### Ethics statement

Both Study 1 and Study 2 conformed to the Code of Ethics of the American Psychological Association, to the Declaration of Helsinki, and to national guidelines. Written, informed consent was obtained from all participants. The study was approved by the ethics committee of the University Osnabrück.

#### Participants

Thirty-six students (majoring in Psychology and Cognitive Science) participated in the study for course credits. Ages ranged from 18–30 years with a mean of 21.78 (SD = 2.47; 19 female participants). They had normal or corrected-to-normal visual acuity and were naive to the purpose of the study as well as to the experimental setup. All subjects signed a written consent form to participate in the experiment.

#### Stimuli

In Study 1 participants observed neutral target images embedded into different emotional contexts (negative, neutral, and positive). Hence, we used four sets of complex images in this study: (1) positive images constituting an emotional context of positive valence, (2) negative images constituting a corresponding emotional context, (3) fractal images constituting a neutral emotional context, and (4) neutral target images showing nature landscapes.

Overall 80 pictures from the international affective picture system (IAPS [40]) served as emotion-laden stimuli (also denoted as “primes”) and were used to influence participants' emotional states. Positive and negative IAPS images were selected with respect to high differences in valence ratings comparable to previous studies [41], [42], [43]. Thereby, 40 IAPS pictures with low valence values (all images <3 on a scale ranging from 0–9; image numbers in the IAPS are: 2053, 2205, 2345.1, 2456, 2683, 2688, 2703, 2710, 2800, 2900, 3180, 3181, 3230, 3300, 3350, 3500, 3530, 3550.1, 6212, 6315, 6520, 6540, 6550, 6821, 8485, 9041, 9075, 9140, 9163, 9181, 9183, 9184, 9187, 9250, 9254, 9332, 9410, 9414, 9419, 9921) served as negative emotional primes and created a negative context for embedded, neutral target images. The positive-context condition was developed using 40 IAPS pictures with high valence values (>7; image numbers in the IAPS: 1340, 1441, 1460, 1500, 1604, 1610, 1630, 1710, 1721, 1722, 1750, 1920, 1999, 2035, 2045, 2057, 2070, 2080, 2156, 2165, 2222, 2260, 2274, 2300, 2311, 2314, 2332, 2341, 2345, 2360, 2370, 2388, 2391, 2530, 2540, 2550, 2650, 2660, , 4599, 4641), excluding pictures showing erotic content. Negative and positive primes differed in their mean arousal ratings [negative primes: M = 5.90, SD = 0.727; positive primes: M = 4.419, SD = 0.642; t-test: p<.001].

For neutral target images we used 30 complex nature images from the McGill Calibrated Colour Image Database [44] depicting bushes, trees, or meadows without man-made objects. These images have been used in previous studies [10], [13], [21], [45].

In order to build a neutral emotional context, we used 40 fractal images that were borrowed from the online fractal database, chaotic n-space network (http://www.cnspace.net/html/fractals.html). This choice is important from two aspects:

First, nature images themselves are not suitable to build an appropriate neutral context, because they are identical to the type of target images we used. In contrast, the emotion-laden IAPS images (i.e., primes) in the positive and negative context conditions substantially differ from nature target images. Hence, a potential oddball-effect could be present in the positive and negative context conditions [46]. This is, when a unique stimulus (i.e., the oddball stimulus; e.g., a flower) is embedded in a train of repeated or similar stimuli (e.g., humans or animals as depicted in IAPS images), the oddball stimulus can seem to persist for a longer duration than the stimuli constituting the context. As a consequence, such an oddball effect could affect eye-movement parameters. Therefore, the primes constituting the neutral emotional context also have to be different from the target images to rule out potential context-dependent differences in viewing behavior that are signatures of oddball vs. non-oddball effects.

Second, the type of neutral primes should be of similar complexity compared to emotion-laden primes, and they should differ significantly in their visual appearance. Hence, monochrome or white-noise images are not appropriate. Even pink-noise images containing natural second-order statistics [47], [48] are not suitable, because they evoke fatigue in the observer and produce viewing behavior differing significantly from what can be found on complex scenes of other categories [21]. Consequently, from the current insights in viewing behavior of complex scenes, fractal images are the first choice to build a neutral emotional context. All images were scaled down or cropped to a resolution of 1280×960 pixels (4∶3) and converted to bitmap format.

#### Apparatuses

The laboratory and experimental setup were identical to those realized in our recent studies [10], [21] in order to allow the comparison of present data with the viewing behavior previously observed. For a detailed description of the system setup, see [21]. In short: the experiment was conducted in a darkened room, providing constant background lighting conditions. Stimuli were presented on a 21-inch Samsung SyncMaster 1100 DF 2004 CRT Monitor (Samsung Electronics, Seoul, South Korea). The display resolution was 1280×960 pixels, the refresh rate was 85 Hz, and the distance to the screen was set at 80 cm without headrest to facilitate normal viewing conditions. The computer running the experiment was connected to the host computer (Pentium 4, Dell Inc., Round Rock, TX, USA) with EyeLink software via a local network.

Eye movements were recorded via the EyeLink II system (SR Research, Ontario, Canada), which uses infrared pupil tracking at a sampling rate of 500 Hz and compensates for head movements. Spatial resolution is better than 0.01° visual angle. To calibrate, participants made saccades to a grid of 13 fixation spots on the screen, which appeared one by one in a random order. Tracking of the eye, giving the lower validation error, started as soon as this value was below 0.35°. After each stimulus presentation, a fixation spot appeared in the middle of the screen to control for slow drifts in measured eye movements. Calibration and validation were repeated in cases of an error larger than 1°. Fixations and saccades were detected and parameterized automatically by the eye-tracker. The first fixation of each trial was excluded from analysis, because its localization was determined by the preceding fixation spot used for drift correction.

#### Procedure

Participants first were informed about the study and had to sign a consent form. Specifically, subjects were explicitly informed about the “unpleasant” stimuli used in the experiment. The possibility to end the experiment whenever desired was acknowledged by signing an additional consent form. Then, all participants had to pass the Ishihara Test for Color Blindness [49].

**Figure 1:**
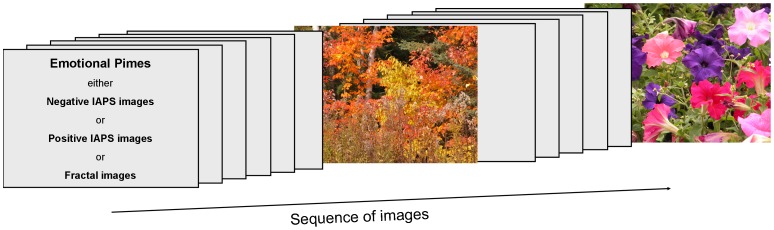
Sequence of images within an emotional context condition. The emotional context was built by positive or negative IAPS images, or by neutral fractal images. Primes were presented in a block intermingled by nature target images, whereby the occurrence of target images was unpredictable.

After the calibration and validation procedures described above, the eye-tracking session proper started. The sequence of emotional contexts (negative, neutral, and positive) was counterbalanced across subjects. Moreover, the sequence of primes constituting these emotional contexts was randomized within each context. Finally, each of the 30 neutral target images was assigned equally often to each of the three emotional contexts, whereby the order of target images within a context was randomly generated for each participant. Besides all these balancing arrangements preventing sequence effects, we also pseudo-randomly varied the position of primes and targets within a block so that a target image followed after three to six primes (see Fig. 1 for an exemplary sequence). As a result, the occurrence of target images was unpredictable, and expectation effects on viewing behavior can be ruled out.

Furthermore, the minimum number of three primes (on average four primes) preceding a target provided an emotional priming of adequate intensity. Previous studies showed that the effect of high-valence IAPS images on papillary responses, heart rate, and skin conductance [43], as well as on ERP amplitudes [50], is significantly different from the effect of neutral images and that these differences were sustained for most of the presentation duration of images. In contrast to these previous studies, several IAPS images were successively presented before target onset in the present study, so that the effect of single IAPS primes should be much more sustainable and, hence, create an intensive emotional context. In accordance with previous studies [41], [42], [43], [50], the presentation duration of each picture was six seconds. This duration ensured direct comparability to our previous eye-tracking studies with free-viewing tasks [10], [21]. At the end, subjects were informed about the purpose of the study.

#### Saccade parameters

Eye-tracking data were analyzed to investigate the fixation duration and the visual step sizes by means of saccade lengths. For all subsequent analyses, the first fixation was excluded, since it was solely a result of the preceding fixation spot used for drift correction. Moreover, in order to prevent data biases, we excluded fixation durations that were shorter than 40 ms or that were more than two standard deviations above the grand mean. The lower temporal bound of 40 ms was chosen in accordance with the literature [1], [51]. The fixation duration was calculated online by the eye-tracking system. Saccade frequency was computed as the number of valid saccades per time unit. Saccade length was operationalized by the Euclidean distance between two consecutive fixations marked by their two-dimensional coordinates in image space. To prevent data biases in the saccade length computation, we excluded all saccades that led to fixation locations beyond the screen boundary.

#### Fixation distribution analysis

To investigate the spread of fixation distributions independent of specific geometrical arrangements, we employed the concept of entropy by following a technique applied recently [21], [52]. An entropy value quantifies the spread of a given fixation distribution without any prior assumption about the geometric properties of the resulting distribution. Higher values indicate a more spread out distribution. The minimum value occurs for a singular distribution; the maximum for a flat distribution. The absolute entropy values, however, depend on image resolution as well as on the size of the Gaussian kernel used for convolution and are not relevant in the present context. For an example of the effect size indicated by numerical differences in entropy values, please see [52]. Hence, for the present purpose, entropy is a suitable signature of the explorativeness of an observer's viewing behavior.

Importantly, estimators of entropy are influenced by sample size [53], [54]. As no general unbiased estimator is available, we equalized the bias using a bootstrapping technique [6], [21], [52]. We analyzed the spread of fixation distributions on a fine-grained level (i.e., single subjects observing single images) as follows: the fixation distribution map of subject *s* viewing image *i* was convolved with a Gaussian kernel, resulting in a fixation density map (FDM). The full width at half maximum (FWHM) of the Gaussian kernel defining the size of the patch was set to 1° of visual angle. In order to address the link between fixation numbers and entropy values, we used a bootstrapping correction by randomly sampling nine fixation points out of the pool of all fixations made by subject *s* on image *i*. Then the entropy *E* of the resulted FDM was calculated according to
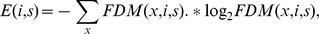
where *x* indicates the position in image space. This procedure was done with 100 repetitions, and finally, the mean entropy was calculated for subject *s* observing image *i*. These bootstrapping-corrected entropy values were subsequently averaged across all images of a category (negative, neutral, or positive primes, as well as target images). All cases in which subjects scanned single images with less than 9 fixations (<5%) were excluded from this bootstrapping analysis to prevent a loss of statistical power and to allow a direct comparison with our previous study [21].

#### Statistical testing

In all statistical tests we chose a significance level of 5% and applied Bonferroni-correction where appropriate. Before applying parametrical tests, Kolmogorov-Smirnov tests were calculated to check normal distribution of data, and homogeneity of variances was tested by means of Levene's test. Effect sizes as indicators of practical significance are reported by partial eta squared η_p_^2^ [0.01 = small; 0.06 = medium; 0.14 = large] in the case of an analysis of variance (Greenhouse-Geisser applied) and by Cohen's d [0.3 = small; 0.5 = medium; 0.8 = large] in the case of a t-test [55].

### Results

#### Viewing behavior on emotional primes

**Figure 2:**
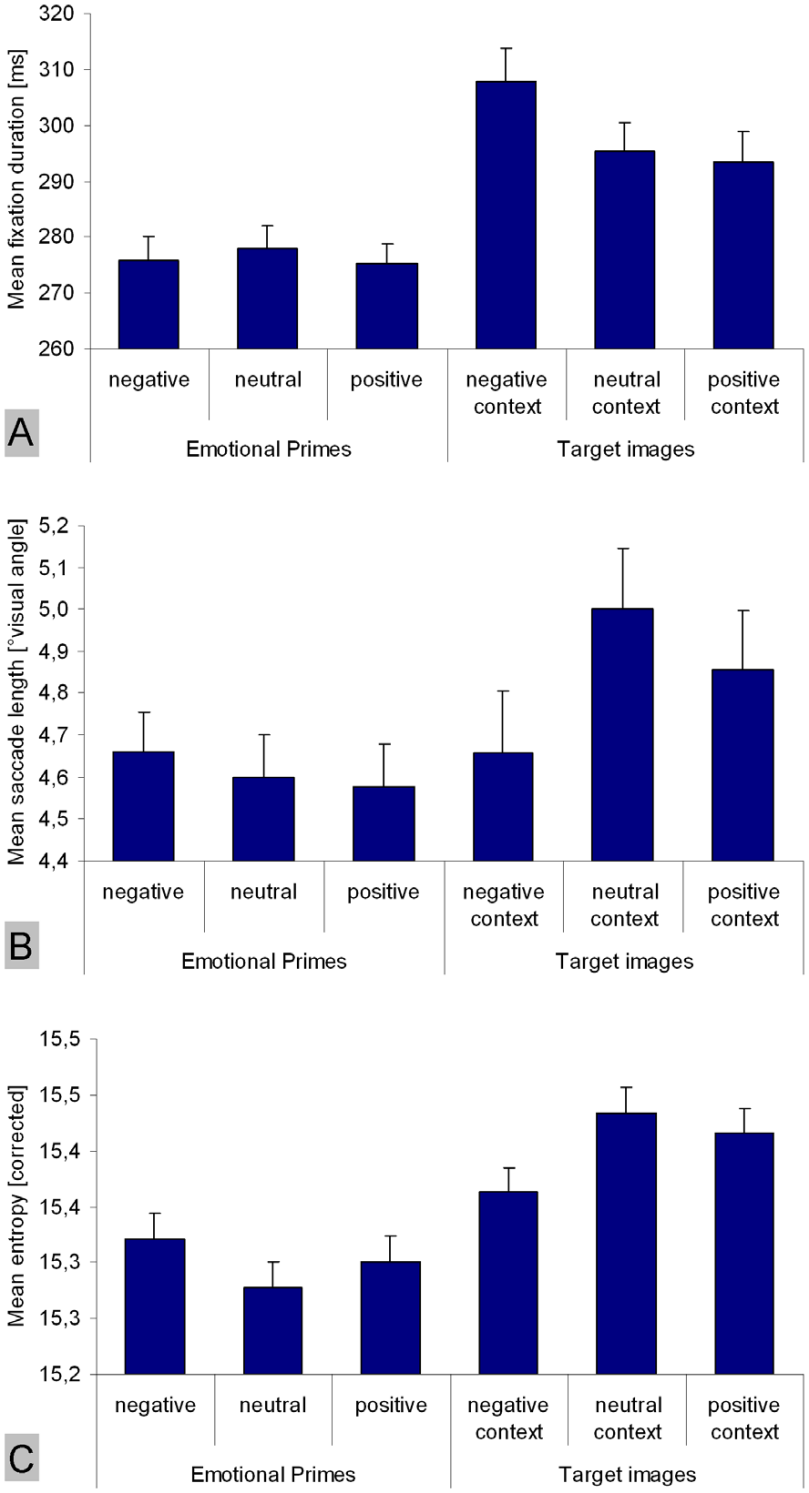
Differences in eye-movement parameters between emotional primes (negative, neutral, and positive) and targets embedded into corresponding emotional contexts. (A) mean fixation duration, (B) mean saccade length, and (C) mean entropy quantifying the spread of fixation distributions. On the left side of each figure, emotional primes are contrasted. On the right side, nature target images embedded into different emotional contexts are contrasted. Vertical lines on top of bars indicate standard error of the mean.

First, we analyzed eye-movement parameters by computing analyses of variance (ANOVA) for repeated measures in order to compare negative, neutral, and positive primes. Eye-movement parameters were normally distributed in all conditions as revealed by Kolmogorov-Smirnov tests [all p≥0.561]. With respect to the mean fixation duration (averaged across all fixations made on a single image), no difference was found between positive IAPS images, negative IAPS images, and neutral fractal images [F(1.869, 65.419) = 0.398; p = 0.659; η_p_^2^ = 0.011] (Fig. 2-A). Regarding the mean saccade length on images, we also found no effect of the prime category [F(1.803, 63.094) = 0.271; p = 0.741; η_p_^2^ = 0.008] (Fig. 2-B). Finally, no dependence of entropy on prime categories was found [F(1.952, 68.304) = 0.843; p = 0.432; η_p_^2^ = 0.024] (Fig. 2-C).

We additionally investigated if the similarity in saccade length and fixation duration between prime types was stable across the observation time of an image. For that purpose, the duration of fixations was scrutinized for the first nine valid fixations as well as for the length of the first nine valid saccades (only <5% of trials showed a fixation number below nine and, hence, were excluded from this analysis). Eye-movement parameters on primes were normally distributed independent of the current fixation or saccade, respectively [all p≥0.260].

**Figure 3:**
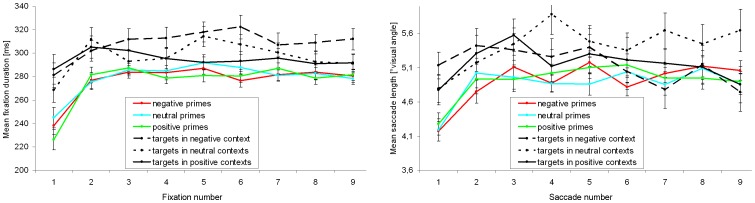
Time dependency of fixation duration and saccade length. Fixation duration (left side) and mean saccade length (right side) for negative, neutral, and positive primes as well as for nature target images depending on the emotional context in which they were embedded. Vertical lines on data points indicate standard error of the mean.

A 3×9 ANOVA (prime type×fixation number) for repeated measures revealed a main effect of the current fixation [F(5.269, 184.417) = 45.811; p<0.001; η_p_^2^ = 0.567] with a large increase from the first to the second fixation [Bonferroni-adjusted t-test: p<0.001]. The fixation duration remained constant from the second fixation as revealed by pairwise comparisons between fixations [all p≥0.284] (see Fig. 3, left side). Again, no effect of the prime type on fixation duration was found [F(1.883, 65.895) = 0.387; p = 0.668; η_p_^2^ = 0.011]. Moreover, no significant interaction between the current fixation and the prime type was revealed [F(9.460, 331.101) = 1.624; p = 0.103; η_p_^2^ = 0.044]. With respect to the length of the first nine valid saccades, a 3×9 ANOVA (prime type×saccade number) revealed a significant change over time [F(6.385, 223.485) = 12.892; p<0.001; η_p_^2^ = 0.269], whereby only a significant increase from the first to the second saccade caused this effect [p<0.001]. Saccade lengths remained constant from the second saccade [all p>0.999] (Fig. 3, right side). Moreover, neither an effect of the prime type was found [F(1.852, 64.806) = 0.072; p = 0.919; η_p_^2^ = 0.002], nor an interaction [F(10.293, 360.242) = 1.097; p = 0.363; η_p_^2^ = 0.030].

To conclude, the primes constituting the emotional contexts did not evoke different viewing behavior by means of fixation duration, visual step size, or spatial explorativeness.

#### Viewing behavior on target images

In the second step, we analyzed eye-movement parameters on target images embedded into the different emotional contexts. Eye-movement parameters on targets were normally distributed in all context conditions [Kolmogorov-Smirnov tests: all p≥0.238].

One-way ANOVAs for repeated measures revealed a significant influence of the emotional context on how participants scanned the target images. With respect to fixation duration, a significant effect of emotional priming was observable [F(1.896, 66.374) = 6.645; p = 0.003; η_p_^2^ = 0.160] (Fig. 2-A). Subsequently, we calculated Bonferroni-adjusted t-tests to pairwisely compare the priming conditions: target images were observed with fixations of longer duration in the negative context compared to the neutral and positive emotional contexts [both p≤0.035]. The neutral and positive contexts did not differ in their influence on target images [p>0.999]. With respect to the mean saccade length on target images, we also found a significant effect of the emotional context [F(1.810, 63.355) = 3.870; p = 0.030; η_p_^2^ = 0.100], whereby the negative emotional context evoked shorter saccades than the neutral context [p = 0.053] (Fig. 2-B). No difference was found between the positive context and the other two priming conditions [both p≥0.401]. Finally, an effect of the priming conditions was found regarding entropy [F(1.875, 65.634) = 4.392; p = 0.018; η_p_^2^ = 0.111] (Fig. 2-C). Thereby, target images presented within the negative context were scanned less extensively than target images embedded into the neutral context [p = 0.033]. Again, no difference was found between the positive context and the other two priming conditions [both p≥0.160]. Consequently, the increased fixation duration and reduced saccade length on target images evoked by the negative context were paralleled by lowered entropy.

As done for the primes, we also analyzed viewing behavior on target images on the level of single fixations and saccades. Fixation durations and saccade lengths were normally distributed in all conditions [Kolmogorov-Smirnov-tests: all p≥0.087].

A 3×9 ANOVA (emotional context×fixation number) for repeated measures revealed a main effect of the current fixation [F(5.652, 197.834) = 3.550; p = 0.003; η_p_^2^ = 0.092]: the duration of the first fixation was significantly shorter than several subsequent fixations [three p≤0.021], which themselves did not significantly differ from each other [all p>0.999] (Fig. 3, left side). Also when limited to the first nine fixations, a significant effect of the emotional context on target observation was found [F(1.887, 66.047) = 5.378; p = 0.008; η_p_^2^ = 0.133]. Bonferroni-adjusted pairwise comparisons of emotional contexts showed shorter fixations on targets embedded into a negative emotional context compared to a positive context [p = 0.013], as well as (by trend) compared to a neutral context [p = 0.081]. No interaction between the current fixation and the emotional context was found [F(9.772, 342.033) = 1.233; p = 0.270; η_p_^2^ = 0.034]. This is the context effect sustained over time, though it was revealed with a short temporal delay, as shown by Fig. 3 (left side).

With respect to the length of the first nine valid saccades, a 3×9 ANOVA (emotional context×saccade number) revealed a change over time [F(6.421, 224.725) = 2.164; p = 0.043; η_p_^2^ = 0.058]. Thereby, the length of the first saccade was minimal and differed significantly from the third saccade [p = 0.021], which had a maximal average length. After the third saccade, the lengths continuously decreased, but Bonferroni-adjusted pairwise comparisons of saccade numbers did not reveal further significant differences [all p≥0.113] (Fig. 3, right side). Furthermore, an effect of the emotional context was revealed by trend [F(1.920, 67.204) = 0.086; p = 0.086; η_p_^2^ = 0.069], with longer saccades on target images embedded into the neutral context, in contrast to the positive and negative contexts. No significant interaction was found [F(10.509, 367.829) = 1.479; p = 0.141; η_p_^2^ = 0.041], and hence, the context effect was relatively constant over time.

To sum up, although different prime types were identically explored, target images embedded into the emotional contexts were observed differentially, depending on the prime type. Hence, no simple transfer effect from the primes to the targets was found on the level of eye movements; rather, viewing behavior on targets seemed to be influenced by the effect of the emotional context on the inner state of the observer. Thereby, the context effect on fixation duration and saccade length was revealed with a short temporal delay after stimulus onset, but was sustained over time.

#### Differences between emotion-laden primes and neutral target images

In the last step, we compared the emotion-laden primes (negative, neutral, and positive) with the target images presented in three different emotional contexts regarding fixation duration, saccade length, and entropy. Fig. 2 depicts the corresponding means. Because of multiple testing, we refer here to a Bonferroni-adjusted significance level of 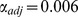.

Overall, fixation durations were significantly shorter on emotion-laden primes (independent of prime type) compared to neutral target images embedded into the three different emotional contexts, as revealed by t-test for paired samples [all p≤0.001; all d≥0.672]. Moreover, saccade lengths were longer on targets embedded into a neutral context compared to primes independent of the primes' emotional category [all p≤0.006; all d≥0.522]. Targets presented in a positive context were also scanned with longer saccades than neutral and positive primes [both p≤0.027; all both≥0.406], but targets in a positive context did not differ from negative primes with respect to saccade length [p = 0.150; d = 0.255]. No difference was found between prime types and targets embedded into a negative emotional context [all p≥0.564; all d≤0.102]. Finally, entropy values were higher on target images than on primes independent of the emotional context and the prime type [all p≤0.006; all d≥0.493], except the comparison of positive primes vs. targets in a negative context [p = 0.046; d = 0.345] and except a non-significant difference between negative primes and targets embedded into them [p = 0.187; all d = 0.255].

To sum up, primes were scanned with fixations of shorter durations, with particularly shorter saccades, and were less spatially extensive in terms of entropy. Consequently, participants observed the primes more actively, but less spatially extensively.

### Discussion of Study 1

Study 1 investigated two mostly disregarded aspects of overt visual attention: (1) Do complex visual scenes differing in their emotional content elicit discriminative viewing behavior, and (2) does the current emotional context specifically affect how observers explore subsequently presented nature images? To answer these questions, neutral target images depicting nature landscapes were embedded in a train of emotion-laden (negative or positive) or neutral primes. Participants were instructed to freely observe the images. We report three central results:

1. The primes constituting a specific emotional context were scanned in a standard fashion. That is, independent of the primes' emotional content, they were observed with fixations of identical duration, with saccades of identical length, and equally spatially extensively.
2. Overall, the primes were scanned more actively but less spatially extensively than the target images. Our participants used a higher saccade frequency to scan the primes (i.e., fixations of shorter duration). Interestingly, the speeded up pace neither corresponded to a more spread out fixation distribution nor to a longer visual step size. Previous studies have already shown that a higher pace of saccades is not necessarily accompanied by a more visually extensive image scanning or longer saccades, respectively [21], [52]. Participants showed a kind of active tunnel vision on primes, independent of prime type. Perhaps the fact that similar primes were presented in a train evoked a characteristic reduction of the focus of attention; however, thereby, the viewing activity was on a high level, which contradicts the hypothesis that participants had been bored by the repeating image type. In a recent study [21] we found that boredom, as elicited by the repetition of pink-noise images, was expressed by a significantly reduced viewing activity paralleled by a tunnel vision. Probably the target images embedded into the sets of primes appeared as unique stimuli (“oddballs,” according to Pariyadath and Eagleman [46]) and hence evoked a more explorative viewing behavior than the repeated prime category. It is possible that the cognitive affordances to process the occasional target images were enhanced in contrast to the similar primes. This interpretation is supported by a study of Velichkovsky, Sprenger, and Pomplun [56], which found a link between the increase of fixation duration and a rise of engagement in cognitive processes. This difference between primes and targets is very interesting, since even the primes differ substantially in their content. However, similarity of images seems not to be limited to the gist of visual scenes, but rather the emotional content could be a strong promoter of the similarity of complex scenes. In any case, the missing difference in viewing behavior on different primes facilitates the interpretation of eye movements on target images.
3. Viewing behavior on neutral target images embedded into the sets of primes was significantly affected by the current emotional context. When target images were shown within a train of negative primes, the viewing behavior was slowed down and less spatially extensive, as indicated by longer fixation durations, shorter saccades, and lower entropy in contrast to a neutral emotional context. The impact of the positive emotional context was in between with respect to saccade length and entropy (except regarding fixation duration) and did not statistically differ from the impact of the neutral and negative emotional contexts. Moreover, the context effect on fixation duration and saccade length was revealed with a short temporal delay after stimulus onset, but was sustained over time, as shown by a more detailed analysis on the level of single fixations and saccades.

All in all, these results clearly indicate that no simple transfer effect from the primes to the targets occurred, but rather viewing behavior on targets seemed to be influenced by the effect of the current emotional context on the inner state of the observer.

## Study 2

The design of Study 1 was optimized to first demonstrate a potential effect of emotional priming on the observer's viewing behavior on neutral target images. The results demonstrate the viability of this concept; however, further questions arose: can we find an effect of emotional priming even when the intensity of priming is reduced? In Study 1 a target image was on average preceded by 4 primes. In Study 2 we reduced the priming to a single image. Moreover, what effect does the type of target image have on eye-movement parameters? In Study 1, the neutral target images were limited to landscape scenes without man-made objects. However, previous studies showed significant differences in viewing behavior between several types of complex scenes [10], [13], [21], [45], [57]. Therefore, we extended the set of nature target images by two further target types in order to reveal potential effects of the target category on eye-movement parameters. Finally, does the post-prime position of target images, i.e., the number of intervening neutral targets, influence the way in which they are observed? Therefore, we conducted a follow-up study that addressed all of these issues in terms of a more elaborate experimental design. In more detail, we investigate the following questions:

1. Can we replicate the non-existing effect between negative and positive emotion-laden images (i.e. primes) on viewing behavior within a new experimental design?
2. Do single, emotion-laden images have a measureable priming effect on neutral target images compared to the more intense priming in Study 1?
3. Do emotion-laden primes of high valence differ from neutral target images regarding eye-movement parameters, as shown in Study 1?
4. Does the post-prime position of neutral images influence the way in which the images are observed?
5. Finally, which impact does the category of the neutral target images have in a certain emotional context constituted by emotion-laden primes? Can we replicate the signature of eye-movement parameters on complex images differing in scene gist [21]? In our previous study, participants repeatedly observed complex images of different categories. In the first presentation run, all images were presented once and without an emotional context. Hence, this first presentation run served as baseline condition that gave us the opportunity to compare these data with the present ones.

### Method

#### Participants

Twenty-four students (Psychology and Cognitive Science) participated in the study for course credits. Ages ranged from 18–32 years with a mean of 23.67 (SD = 3.76; 16 female participants). All subjects had normal or corrected-to-normal visual acuity, and they were naive to the purpose of the study as well as to the experimental setup. A written consent form to participate in the experiment was signed by all subjects. As in Study 1, ethical standards of the APA, the German Psychological Society (DGPs) and the German Psychological Association (BDP) were considered.

#### Apparatuses and stimuli

The laboratory and experimental setup in Study 2 was identical to those used in Study 1. Overall, 36 IAPS images were used as emotional primes to influence the participants' emotional states. All negative images had a valence value below 3 (image numbers in the IAPS: 2683, 2811, 3530, 6212, 6313, 6350, 6520, 6550, 6570, 6821, 8485, 9163, 9183, 9187, 9254, 9410, 9414, 9921); all positive images were rated with 7 or higher (image numbers: 1441, 1460, 1710, 1750, 1999, 2045, 2057, 2070, 2080, 2260, 2274, 2311, 2347, 2530, 2540, 2550, 2650, 2660). Negative and positive primes differed in their arousal ratings [negative primes: M = 6.584, SD = 0.367; positive primes: M = 4.531, SD = 0.500; t-test: p<.001]. No neutral primes were included.

**Figure 4:**
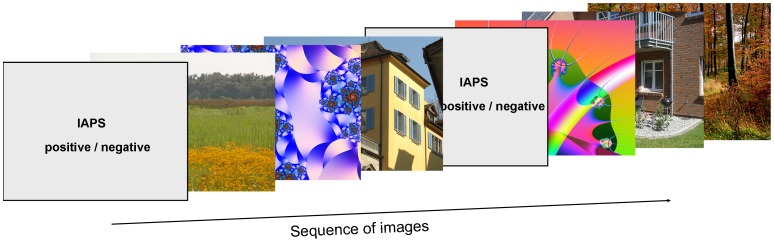
Sequence of images (example). One positive or negative IAPS image serving as prime was followed by three neutral target images belonging to the three different image categories (nature, urban, fractal), respectively. The emotional content of primes as well as the order of target images were randomized.

One hundred and eight complex images formed the set of neutral target stimuli. See Fig. 4 for examples. They belonged to three categories: the first category (nature) contained 36 images from of the McGill Calibrated Colour Image Database [44], as in Study 1. The second category (urban) comprised 36 images, such as streets, vehicles, and house exteriors. These pictures were taken with a high-resolution camera (Nikon D2X) and were unfamiliar to the participants. Nature and urban images were free of people or writing. The third category (fractal) consisted of 36 software-generated fractal pictures taken from the online fractal database, chaotic n-space network (http://www.cnspace.net/html/fractals.html). Four, eight, and eight images of the nature, urban und fractal categories, respectively, were also used in previous studies [10], [21]. All images from these three categories were scaled down or cropped to a resolution of 1280×960 pixels (4∶3) and converted to bitmap format.

#### Procedure

Participants were first informed about the study and signed a consent form. Subjects were explicitly informed about “unpleasant” stimuli used in the experiment. Then, they had to pass the Ishihara Test for Color Blindness [49]. After the common calibration and validation procedure, the eye-tracking session started. The emotional IAPS images (denoted as “primes”) were presented randomly. In this way, participants were not able to predict the following prime type. After one prime, three neutral target images were presented, whereby each target image derived from another category (nature, urban, or fractal), but their order was randomized (see Fig. 4). To prevent any potential bias, the sequence of images was balanced within individual subjects and across all subjects. As a consequence, each target image occurred equally often at a certain post-prime position, while also considering the emotional content of the preceding prime. All images were presented for 6 seconds.

#### Data analysis

The analysis of fixation duration, saccade frequency, and entropy was identical with the procedure applied in Study 1 as described above.

### Results

#### Viewing behavior on emotion-laden images: positive versus negative primes (question 1)

**Figure 5:**
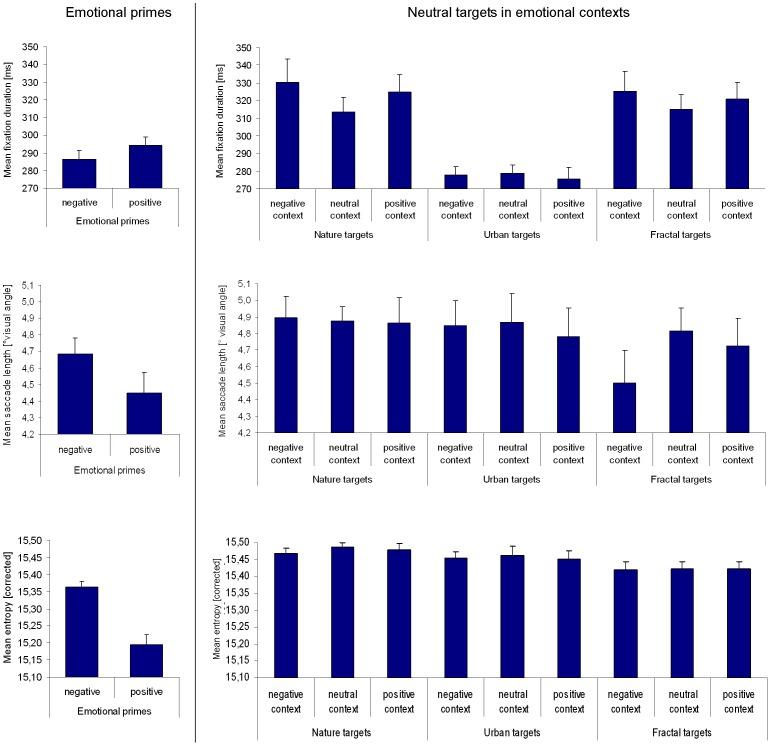
Eye-movement parameters on emotion-laden and neutral images. Eye-movement parameters on negative and positive IAPS images serving as emotion-laden primes (left side) and parameters on neutral target images (nature, urban, and fractal) depending on target type and emotional context in which target images were embedded (right side). Vertical lines above bars indicate standard error of the mean. Note: The axis of ordinates differs between primes and targets with respect to the scaling. Moreover, the experimental setups of Studies 1 and 2 were identical, and therefore, absolute parameter values are comparable between both studies.

Viewing behavior on negative and positive primes was statistically compared by t-tests for paired samples. All eye-movement parameters were normally distributed [Kolmogorov-Smirnov tests: all p≥0.639]. With respect to fixation durations we found a small- to medium-sized difference: positive IAPS images, compared to their negative counterparts, elicited significantly longer fixations by trend [t(23) = 1.995; p = 0.058; d = 0.407] (Fig. 5, left side). This difference was paralleled by shorter saccades used to scan positive primes [t(23) = −2.516; p = 0.19; d = 0.514,] and by the mean entropy of fixations made on a single image [t(23) = −9.128; p<0.001; d = 1.865], indicating a less extensive spatial exploration of positive primes in contrast to negative ones (Fig. 5, left side). Consequently, negative IAPS images were scanned more actively and spatially extensively than positive images.

**Figure 6:**
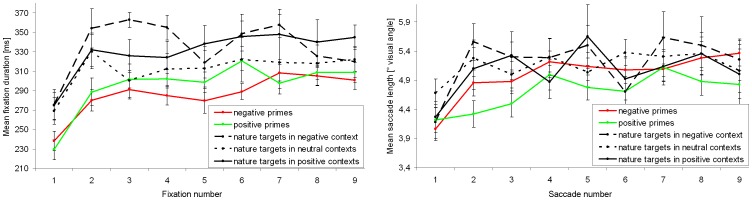
Time dependency of fixation duration and saccade length. Fixation duration (left side) and mean saccade length (right side) for negative and positive primes as well as for nature target images depending on the emotional context in which they were embedded. Vertical lines on data points indicate standard error of the mean.

As in Study 1, we additionally scrutinized the duration of fixations for the first nine valid fixations, as well the length of the first nine saccades [Kolmogorov-Smirnov tests: all p≥0.157]. With respect to fixation duration, a 2×9 ANOVA (prime type×fixation number) for repeated measures revealed a main effect of the current fixation [F(5.607, 128.956)  = 13.479; p<0.001; η_p_^2^ = 0.370]. We again found longer fixations on positive primes compared to negative primes by trend when analyzing only the first nine fixations [F(1, 23) = 3.429; p = 0.077; η_p_^2^ = 0.130]. No significant interaction was revealed [F(5.546, 127.553) = 1.164; p = 0.330; η_p_^2^ = 0.048], though Fig. 6 (left side) shows that the difference between positive and negative IAPS images increased with the current fixation number. With respect to the length of the first nine valid saccades, a 2×9 ANOVA (prime type×saccade number) revealed a significant increase in saccade length over time [F(5.460, 125.571) = 5.073; p<0.001; η_p_^2^ = 0.181], longer saccades on negative IAPS images [F(1, 23) = 7.174; p = 0.013; η_p_^2^ = 0.238], but no significant interaction [F(6.251, 143.780) = 0.684; p = 0.669; η_p_^2^ = 0.029]. The first saccade, however, did not differ between positive and negative primes (Fig. 6, right side).

#### Viewing behavior on neutral target images depending on the emotional context (question 2)

In order to answer whether, compared to Study 1, less intensive emotional priming in Study 2 also specifically affected viewing behavior on neutral target images, a 3×3 ANOVA (emotional context×target type) for repeated measures was calculated for all three eye-movement parameters [Kolmogorov-Smirnov tests: all p≥0.258]. This analysis simultaneously addressed research question 5 (the impact of target type on viewing behavior). As no interaction was found with regard to any of these parameters [all F(3.163, 72.758) ≤1.368; all p≥0.259; all η_p_^2^≤0.056], only the main effect of the emotional context is reported in this section and the main effect of the target type will be reported in the result section addressing research question 5.

With respect to the mean fixation duration on neutral target images embedded into different emotional contexts (negative, neutral, and positive), we found a medium-sized, but not statistically significant, difference between the emotional contexts when fixation durations were averaged across target types [F(1.564, 35.974) = 2.256; p = 0.130; η_p_^2^ = 0.089]. This context effect was mostly driven by an effect on nature targets as revealed by a one-way ANOVA [F(1.885, 43.349) = 2.635; p = 0.086; η_p_^2^ = 0.103], whereby the negative context evoked longer fixations than the neutral context, as was also found in Study 1 (compare Fig. 5, right side, and Fig. 2). No context effect was found for urban and fractal images [both F≤1.172; both p≥0.314; both η_p_^2^≤0.048].

With respect to the duration of the first nine fixations made on target images [Kolmogorov-Smirnov tests: all p≥0.113], the context effect for nature images became statistically significant, as revealed by a 3×9 ANOVA (emotional context×fixation number) [F(1.829, 42.076) = 3.455; p = 0.045; η_p_^2^ = 0.131] (Fig. 6, left side). In contrast, on urban and fractal images no effect of the emotional context on fixation duration was revealed for the first nine fixations [both F≤0.777; both p≥0.444; both η_p_^2^≤0.033] (not depicted). Moreover, no interaction between the emotional context and the fixation number was found for any target type [all F≤1.199; all p≥0.302; all η_p_^2^≤0.050]. However, fixation durations showed an increase during the trajectory on nature images (Fig. 6, left side), as well as on urban and fractal target images [all F≥3.909; all p≤0.003; all η_p_^2^≥0.145].

With respect to the mean saccade length [Kolmogorov-Smirnov tests: all p≥0.122], no significant effect of the emotional context was revealed by the 3×3 ANOVA (emotional context×target type) i.e. when saccade lengths were averaged across target types [F(1.828, 42.034) = 1.010; p = 0.367; η_p_^2^ = 0.042]. However, one-way ANOVAs for each target type [all F≤1.956; all p≥0.090; all η_p_^2^≤0.100] revealed a medium- to large-sized effect of the emotional context on saccade lengths for fractal images [η_p_^2^ = 0.100] (Fig. 5, right side).

We also analyzed the first nine valid saccades by a 3×9 ANOVA (emotional context×saccade number) for each target type, but neither a main effect of the emotional context was found [all F≤2.131; all p≥0.132; all η_p_^2^≤0.085], nor an interaction between the emotional context and the current saccade [all F≤.984; all p≥0.455; all η_p_^2^≤0.041]. However, the increase of fixation duration from stimulus onset found on nature images was paralleled by an increase of saccade length on nature targets [F(5.830, 134.088) = 2.850; p = 0.013; η_p_^2^ = 0.110] (Fig. 6, right side), but no difference between saccades existed for fractal and urban target images [both F≤1.439; both p≥0.204; both η_p_^2^≤0.059] (not depicted).

With respect to entropy, the 3×3 ANOVA (emotional context×target type) did not show an effect of the emotional context on neutral target images [F(1.673, 38.471) = 0.315; p = 0.693; η_p_^2^ = 0.014] (Fig. 5, right side).

Consequently, the strong effect of the emotional context on fixation durations found in Study 1, which exclusively used nature targets, was also found in Study 2 using less intensive emotional priming. However, the signature of the context effect partially differed from those in Study 1. In contrast to Study 1, the impact of the emotional context (positive or negative) regarding saccade length and entropy was small and not statistically significant, in general.

#### Differences between emotion-laden primes and neutral target images (question 3)

As in Study 1, we compared the emotion-laden primes (positive and negative) with the neutral target images presented in three different emotional contexts regarding fixation duration, saccade length, and entropy. This comparison was done separately for each emotional context in which the target images were presented. Because of multiple testing, we refer here to a Bonferroni-adjusted significance level of 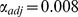. Fig. 5 depicts the corresponding means for each of the five image sets. Fixation durations were significantly shorter on emotion-laden primes (negative and positive) compared to the nature target images embedded into three different emotional contexts [all p≤0.006; all d≥0.625], and hence, the results of Study 1 were replicated. Fixation durations on fractal targets were also longer than on both prime types [all p≤0.007; all d≥0.602]. The effect reversed on urban targets, whereby the differences to positive primes was larger [all p≤0.002; all d≥0.693] than the differences to negative primes [all p≤0.093; all d≥0.357].

With respect to the mean saccade length, nature targets were scanned with longer saccades than emotion-laden primes [all p≤.019; all d≥0.513], except nature targets in positive context vs. negative primes [p = 0.148; all d = 0.305]. Urban target images were scanned with longer saccades than positive primes [all p≤0.008; all d≥0.597], but the mean saccade length did not differ between negative primes and urban targets independent of the emotional context in which they were embedded [all p≥0.167; all d≤0.291]. Fractal targets were scanned with longer saccades than positive primes when targets were presented in a neutral context [p = 0.002; all d = 0.719], but apart from that, no difference in saccade length was found between emotion-laden primes and fractal images [all p≥0.059; all d≤0.407].

Regarding entropy, we found higher values on nature targets (independent of emotional context) than on primes (independent of prime type) [all p≤0.001; all d≥1.246]. Furthermore, urban targets in all emotional contexts [all p≤0.001; all d≥0.985], as well as fractal targets [all p≤0.025; all d≥0.495], were scanned more spatially extensively than negative and positive primes.

To conclude, targets were scanned more spatially extensively and with longer saccades than primes. Saccade frequency, however, was higher on emotion-laden primes in contrast to nature and fractal images, but the effect reversed on urban images.

#### The effect of the post-prime position of neutral targets on viewing behavior (question 4)

The design of Study 2 allowed investigating the effect of the post-prime position of neutral target images on eye-movement parameters [Kolmogorov-Smirnov tests: all p≥0.312]. We first averaged across target types. Subsequently, viewing behavior on targets was analyzed depending on the targets' post-prime position by means of a 2×3 ANOVA (prime type×post-prime position of targets).

The mean fixation duration slightly decreased with an increasing post-prime position of the target; however, it did not reach significance [F(1.657, 38.118) = 2.624; p = 0.095; η_p_^2^ = 0.102]. We found neither an effect of the prime type on fixation duration [F(1, 23) = 1.708; p = 0.204; η_p_^2^ = 0.069], nor an interaction [F(1.716, 39.472) = 0.076; p = 0.902; η_p_^2^ = 0.003].

Regarding the mean saccade length, the ANOVA did not reveal any effect [all F≤1.049; all p≥0.353; all η_p_^2^≤0.044]. Moreover, no effect was found with regard to entropy [all F≤0.888; all p≥0.356; all η_p_^2^≤0.037]. When introducing the target type as an additional factor in the variance analytic design, no moderating effect of target type was revealed. Hence, eye-movement parameters differed significantly between primes and targets (see research question 3 above); however, none of the three eye-movement parameters revealed a significant dependence on the post-prime position of the targets.

#### The effect of target type on viewing behavior in the context of emotional priming (question 5)

In this section, we address the main effect of the target type revealed by the 3×3 ANOVA (emotional context×target type), which was above reported with respect to the main effect of the emotional context on viewing behavior (research question 2).

**Figure 7:**
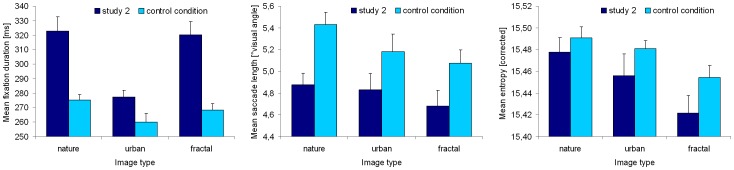
Eye movement parameters on neutral targets of three categories in Study 2 and in our previous study [**21**]. In Study 2, the sequence of target images was intermingled with emotion-laden IAPS images, while in the previous study the targets were presented solely. Note: We only report the results of the first presentation run of [21]. The experimental setup and the analysis of parameters were identical in both studies.

The target type (nature, urban, and fractal) showed a significant impact on the mean fixation duration [F(1.978, 45.503) = 30.241; p<0.001; η_p_^2^ = 0.568], whereby post-hoc t-tests (alpha-adjusted) revealed longer durations on nature as well as on fractal images, in contrast to urban images [both p<.001], but no difference between fractal and nature images [p>0.999]. This result pattern perfectly replicates the effect of the image type on fixation duration as reported before [21] (Fig. 7, left); however, fixation durations were generally longer in the present study, as well as the absolute differences between the image types. Therefore, when presented in the context of emotional stimuli, these target images are observed with lower saccade frequency.

With respect to the mean saccade length on target images, no significant effect of target type was found [F(1.702, 39.136) = 2.082; p = 0.145; η_p_^2^ = 0.083]. The mean saccade length on the targets, however, was generally shorter than in our recent study [21], as shown by Fig. 7 (middle position).

Finally, target types differed significantly regarding entropy [F(1.906, 43.828) = 8.276; p<0.001; η_p_^2^ = 0.265]. Bonferroni-adjusted t-tests for paired samples revealed a more extensive exploration of nature and urban images in contrast to fractal images [both p≤0.046], but no difference between nature and urban images [p = 0.503]. This image-type effect on entropy is a perfect replication of the corresponding effect reported in [21], whereby the absolute entropy values are smaller (see Fig. 7, right).

Consequently, the relative impact of different target types remained even when targets were presented in an emotional context; however, viewing activity was slowed down in general by the emotional contexts, as indicated by longer fixations, shorter saccades, and reduced entropy.

### Discussion of Study 2

Study 2 addresses a total of five questions and replicates and extends results of the effect of emotional priming on viewing behavior, as reported previously in Study 1. The set of nature target images was extended by two further target types, namely, urban and fractal images, to generalize (or not) the conclusions. Additionally, we focused on the potential impact of the targets' post-prime position on viewing behavior, as well as on a comparison with our recent study [21] serving as a control condition here. Several important results are discussed in the following with respect to the research questions (1–5):

(1) The data show that, in contrast to Study 1, negative IAPS images were scanned more actively and spatially extensively than positive images, expressed by shorter fixation durations, longer saccades, and a more spread out fixation distribution. That is, different prime types were not scanned in different ways when presented within a block (Study 1), but negative primes were more actively and spatially extensively scanned than positive primes when presented individually (Study 2). Importantly, 77.8% of the negative primes and 88.9% of the positive primes were identical in both studies, and hence, differences in the stimuli material are not a reason for the varying results between the studies. Rather, this difference supported the idea of a characteristic viewing behavior elicited when similar images are presented in sequence (see discussion of Study 1). In Study 1, no effect of the primes' emotional content was found on exploratory viewing behavior. In Study 2, in contrast, effects on viewing behavior were dependent on the prime type. We speculate that this results from different sensitivities to positive and negative emotional content and a ceiling effect (due to intense priming) in Study 1. The more active exploration of negative IAPS images compared to positive ones (shorter fixations, longer saccades, and higher entropy) supports the classification of IAPS pictures along the arousal axis where positive stimuli elicit less arousal than negative ones [58]. Alternatively, the higher viewing activity found on negative primes may be derived from a faster neuronal processing of visual input, as suggested by van Merle, Hermans, Qin, and Fernández [59] who found that stress amplified sensory processing in early visual regions.

We specified previous findings stating that both negative and positive emotional stimuli are associated with enhanced visual attention processes [28], [29], [30] when presented simultaneously with neutral targets. Highly negative emotional stimuli are possibly of greater emotional relevance, motivated by an underlying defensive system responsible for efficient reactions to harmful stimuli. For this reason, attention to negative stimuli prevails [60]. This result, however, has been specified by research on personality traits, indicating that optimistic people orientate more to positive stimuli [61], and the effect appears not to be stable across the adult's lifespan. Older adults also tend to focus more on positive information [62] and rate differences between good and bad as less severe in specific contexts [63]. Moreover, Mather and Sutherland [64] pointed out that exposure to positive stimuli can broaden cognitive processes, including one's attentional scope. Therefore, no overall advantage for positive or negative stimuli can be derived from present research. Rather, perception of emotional information depends on the individual's current state, which is consistent with related research [21] indicating that personality states and traits are relevant top-down influences in the process of visual attention. Nevertheless, Study 2 shows that, under certain circumstances, the emotional content of images specifically influences the way in which those images are explored.

In this context, the analysis of the first nine saccades and the duration of the respective fixations provided further important insights: in Study 2, the difference between positive and negative primes was not present from stimulus onset, but increased over time. Participants obviously had to recognize the gist and valence of the scene first, which was unpredictable, before switching to a specific viewing behavior.

(2) Moreover, the less intensive emotional priming in Study 2 (compared to Study 1) also specifically affected viewing behavior on neutral target images. Thereby, the effect of the emotional context was small to moderate in general. However, on nature targets, a medium- to large-sized context effect on fixation duration was found partially replicating the corresponding signature in Study 1. Furthermore, a medium- to large-sized context effect on saccade lengths was found on fractal images. Independent of the emotional context, fixation durations increased over time on all target types, but saccade lengths increased after stimulus onset only on nature targets. Interestingly, the spread of fixation distributions, i.e., participants' explorativeness on neutral scenes, was not affected by the emotional context in which they were embedded. Obviously, the emotional priming by single stimuli was too weak to elicit changes in entropy.

(3) In addition to that, fixation durations were shorter on emotion-laden primes compared to nature and fractal target scenes, replicating the result of Study 1. The effect reversed on urban targets. Moreover, all types of neutral targets were scanned with longer saccades than emotion-laden primes, but the effects were larger between targets and positive primes. Finally, entropy values were higher on targets than on primes, independent of the current emotional context and the primes' category. These results are mostly congruent with the corresponding results of Study 1. Obviously, images of high valence elicit a very different viewing behavior than neutral images do. However, in Study 1 fractal images that served as neutral primes were observed just like emotion-laden IAPS images. Once more, this non-effect of prime type probably resulted from the repetition of similar primes within a block, i.e., emotional context.

(4) It is also important to note that these differences between primes and targets in eye-movement parameters remained constant over time, because viewing behavior remained largely constant on targets across three post-prime positions. Only the mean fixation duration on target images slightly decreased with an increasing post-prime position of targets.

(5) Finally, we replicated the impact of target type on viewing behavior previously found [21]. Hence, the impact of image type on viewing behavior seems to be strong, even when emotional priming is introduced. However, viewing activity was slowed down in general by the emotional contexts, as indicated by longer fixations, shorter saccades, and reduced entropy in the present Study 2. This indicates an additional influence of the emotional priming on viewing behavior. Obviously, neutral images were scanned less actively when presented in a context of highly emotional images than when presented alone. This suggests that the reduced viewing activity could be a possible result of interference between the emotional engagement owing to preceding emotional pictures and the processing of the actual neutral target. In other words, even the less intensive emotional priming in Study 2 influenced the emotional state of the observer, which, in turn, prolonged the processing of neutral target stimuli.

## Conclusion

In summary, Study 2 provides evidence for an externally located impact of emotion on viewing behavior under natural conditions [65]. This is the effect of the emotional content of complex scenes (IAPS images) on the way in which they are observed. However, Study 1 showed that this emotion effect diminished when similar images were presented in a train, although image properties did not change. Neutral fractal images, which were used as primes in Study 1, showed the same effect on viewing behavior as high-valence IAPS images. This result is very surprising, but highlights the necessity to consider several context factors that can affect eye movements in a top-down matter. Obviously, the emotional component of stimuli interacts with further context conditions; for example, the density of similar images in a set of images. Future studies should address these and other potential high-level interactions influencing viewing behavior.

In addition to that, we also found evidence for an internally located impact of emotion on how people observe complex stimuli, i.e., the emotional context seems to affect the inner state of the observer, which, in turn, influences eye-movement parameters. Against this background, future studies should further scrutinize the impact of certain emotional states on eye-movement parameters under natural conditions. We conclude that the emotional state of an observer influences his current viewing behavior; however, attention has to be paid to the kind of emotion-related processes that are manipulated in experimental studies. For example, it seems critical to distinguish between emotion and arousal [65]. According to Kensinger [66] (p. 241), “a widely-accepted framework proposes that affective experiences are best characterized in a two-dimensional space.” In one dimension, valence ranges from highly negative to highly positive, and in a second dimension, arousal ranges from calming to exciting. In the present study, IAPS images were selected according to their valence ratings, but arousal values were not considered because of the small number of appropriate images of positive and negative valence that correspond regarding arousal values on the one hand, but which are suitable for eye-tracking studies on the other hand. In the IAPS very few positive images are included that evoke high arousal aside from erotic stimuli, which elicit a highly specific viewing behavior [67]. Hence, it would be fruitful for vision research to create and validate more emotional stimuli that are suitable for eye-tracking experiments in order to disentangle the influences of emotional valence and emotional arousal on viewing behavior under natural conditions.

Both present studies revealed that primes which constitute an emotional context were viewed at an accelerated pace compared to neutral target images (except urban images). Although the emotional effect was long-lasting, viewing of subsequent neutral stimuli exhibited a rebound with decelerated viewing. This deceleration was paralleled by an expanded scope of exploration independent of the intensity of the emotional priming. Moreover, the mean visual step size is enlarged on target images compared to primes.

Finally, the intensity of the emotional context was revealed as a significant factor on viewing behavior under natural conditions. The effect of the emotional priming on the observation of target images was larger overall in the case of a strong emotional context (Study 1). However, the mere presence of emotion-laden IAPS images in a set of different complex scenes (Study 2) slowed down viewing activity in general in contrast to a set of comparable images, but without emotional stimuli [21]. In this context, the remaining effect of the category of target images is also remarkable. All in all, our results suggest that approaches of visual perception and perception in general should focus more on the individual cognitive aspects of these processes, as well as on specific context factors.
